# Views of community health workers on the integration of a physiotherapist into a ward-based outreach team

**DOI:** 10.4102/sajp.v78i1.1645

**Published:** 2022-10-19

**Authors:** Regina Mashole, Lucy Fernandes, Kebogile Mokwena

**Affiliations:** 1 Department of Public Health, School of Healthcare Sciences, Sefako Makgatho Health Sciences University, Pretoria, South Africa

**Keywords:** physiotherapist, ward-based outreach teams, community-based services, rehabilitative services, South Africa

## Abstract

**Background:**

Due to changes in the disease profile and lifestyle of individuals in South Africa, the limited health care facilities available have experienced congestion and overcrowding, affecting health care service delivery. Ward-based outreach team (WBOT) programmes were implemented to strengthen primary health care, improve access and alleviate the congestion occurring at these facilities. However, WBOTs have limitations in terms of medical knowledge and rehabilitative skills.

**Objective:**

To explore the views of community health workers (CHWs) on the integration of physiotherapists into WBOTs.

**Method:**

A qualitative research design making use of focus group discussions (FGDs) was used. Through purposive sampling, 58 CHWs who were members of WBOTs were recruited. The WBOTs were from 10 selected primary health care centres in the Tshwane district, Region 2. Six FGDs were conducted. The audio-recorded data were transcribed verbatim. The transcripts were transported into NVivo 12 for thematic analysis.

**Results:**

The views of the CHWs were that the WBOTs can benefit from having a physiotherapist as a member of the team. The WBOTs do not have adequate skills to attend to the physiotherapy needs of communities. People in the community have challenges in accessing physiotherapy services, and physiotherapy services can enhance the performance of the WBOTs by providing training to the WBOTs and providing clinical services to community members.

**Conclusion:**

Community-based rehabilitative services with a physiotherapist as part of the WBOTs can enhance and strengthen the services of the WBOTs, which can improve the treatment outcomes for communities.

**Clinical implications:**

The WBOTS will be empowered to provide clinical services to the vulnerable people in the community that they serve.

## Introduction

The South African health care system is faced with a quadruple burden of disease (Pillay-Van Wyk et al. 2016). The quadruple burden of disease includes HIV, AIDS, TB, noncommunicable diseases (NCDs), violence and injuries and diseases related to maternal and child health (Basu 2018; Maredza et al. 2016; Modjadji & Madiba 2019). Although many survive the above-mentioned conditions and diseases, some of these diseases may result in limitations in functioning and disability, increasing the need for rehabilitation, which is the key health strategy to address compromised functioning and quality of life for these individuals (World Health Organization [WHO] 2017). Access to rehabilitation services remains a challenge for many persons with disabilities (Hanass-Hancock, Myezwa & Carpenter 2015; Vergunst et al. 2015; Visagie & Swartz 2016), especially those who come from vulnerable communities and have limited financial and health service resources (Gamiet & Rowe 2019). Community-based rehabilitation with transdisciplinary teamwork is important for the improvement of quality management of the needs of vulnerable people in the community (Vyt 2008).

The South African Government initiated the National Health Insurance (NHI) Plan, which was expected to revolutionise health care delivery in the country (Naidoo 2012). The NHI’s focus is to ensure access to health care by subsidising those who cannot afford health care services (South African Government 2022). The adoption of the NHI plan is in line with the implementation of the primary health care (PHC) approach based on the Alma Ata declaration (WHO 1978). Primary health care is a people-centred approach that includes health promotion, disease prevention, treatment, rehabilitation and palliative care dealing with the physical, mental and social well-being of a person throughout their lifetime (WHO 2022). Through PHC and the NHI, all South Africans will receive equal treatment based on their health needs and not based on their socio-economic status (South African Government 2022).

At the centre of the NHI is the plan to strengthen and improve PHC by establishing and implementing ward-based outreach teams (WBOTs) (Khuzwayo & Moshabela 2017), which consist of community health workers (CHWs) with a range of different backgrounds (educational, experiential and training) and competencies resulting in a cadre of workers with varied skill levels, literacy levels and capacities (Vyt 2008). The WBOTs are supported by nurse team leaders and are linked to local PHC facilities (via referral, support and oversight) (Schneider et al. 2018). The WBOTs at a PHC level assist patients to adhere to medication and the management of chronic conditions (Naidoo et al. 2018; Schneider et al. 2018). However, there is evidence showing that the WBOTs lack the skills to address the needs of people with disabilities (Austin-Evelyn et al. 2017; Sherry 2015). Physiotherapy, an important part of rehabilitation for people with disabilities, forms an integral part of services offered at a PHC level (Maleka, Franzsen & Stewart 2008). The problem is that the training and scope of practice of CHWs who form part of the WBOTs have not focused on the social determinants of health or the development of skills required for community mobilisation (Schneider et al. 2018). Working with physiotherapists as part of a community rehabilitation team can empower the WBOTs with basic skills in rehabilitation care in the community. Thus, the aim of our study was to explore the integration of physiotherapists as members of WBOTs.

## Method

In our qualitative, exploratory study conducted in Tshwane, focus group discussions (FGDs) were used to explore the views of CHWs on the integration of a physiotherapist in WBOTs. This design was considered to be appropriate because the potential inclusion of a physiotherapist will impact on how the WBOTs will function, hence the need to explore the views of the CHWs who have been functioning without this inclusion. Our study should provide in-depth information to help to understand the views of the participants on this issue (Gundumogula 2020).

The City of Tshwane is divided into seven regions and has a total of 124 health facilities, comprising clinics, community health centres, district and regional hospitals, central or tertiary hospitals and other hospital groups (private hospital facilities). The settings for our study were 10 PHC facilities of Region 2, Tshwane District. Region 2 is in the northern part of the city, and the borders of the region are the Sinoville-Montana areas in the south, the Temba-Hammanskraal areas in the north, the N1 Highway in the east and the Onderstepoort-Soutpan Road in the west. The townships Stinkwater and Hammanskraal are included in Region 2, which also houses both vast and small agricultural holdings (Cooperative Governance and Traditional Affairs [COGTA] 2020). Region 2 has an estimated population figure of 339 175 people and 115 882 households (Statistics South Africa 2011).

Our study population included CHWs from the Tshwane district, Region 2. There were 31 WBOTs consisting of about 320 CHWs (Tshwane District WBOT presentation 2016). Each WBOT consisted of a team leader and 6–20 CHWs. The team members of the WBOT were recruited by firstly having a formal meeting with each team, explaining the aims and method of our study. The team members who were willing to participate were encouraged to list their names with the team leader, and the first 11 CHWs were chosen. All CHWs who voluntarily consented to participate and who were actively part of a WBOT working in the community were included. CHWs who were still undergoing training, not working actively in the community, not part of a WBOT or who refused to participate were excluded.

### Sampling procedure

The 10 PHC facilities and clinics in Region 2 were numbered, and six were chosen randomly by taking numbers out of a hat. Because of financial and time constraints, only six study sites were chosen. The sample size was limited to one team leader and a maximum of 11 CHWs.

### Data collection tool

An FGD interview guide was developed and was informed by published studies to answer our study question (Schneider et al. 2018). Topics covered were descriptions of the situations encountered in the community where the advice and skills of a physiotherapist could be valuable and how they deal with patients who need treatment that should be provided by a physiotherapist. They were then asked what their views were about the inclusion of a physiotherapist in a WBOT. Sociodemographic information like age, gender and time working as a member of the WBOTs of the CHWs participating in our study were captured in a self-administered questionnaire.

The CHWs were invited to participate in the FGDs after the first author introduced the research topic and aims of our study. On the day of data collection, the focus groups had already been formed by the team leader. Six FGDs consisting of a team leader and a maximum of 11 CHWs were conducted. On average, the FGDs that were conducted by the first author lasted between 30 and 45 min.

### Data analysis

Data obtained from the FGDs were audio-recorded and transcribed verbatim. NVivo (QSR International, Cambridge, MA) version 12 was used for the analysis. The transcripts were read multiple times to initiate the development and definition of codes. To analyse the textual data, qualitative thematic analysis was applied (Maguire & Delahunt 2017). The themes and codes that emerged from the conversations were defined, described and modified continuously to ensure consistency in the coding process applied to all the transcripts. Quotes used were attributed with gender, first letter of the fictious name and the position in the WBOT namely CHE or Outreach Team Leader (OTLs).

### Trustworthiness

Trustworthiness was dependent on credibility, dependability, conformability and transferability.

*Credibility* was accomplished by audio-recording the interview. This was further achieved by making use of relevant probes, obtaining detailed field notes and careful observation and documenting of the nonverbal communication of the participants, as the participants were the only ones who could legitimately judge the credibility of the results. Credibility was also enhanced by the fact that the first author is a physiotherapist without any affiliation with the study population, which could have influenced the responses of the participants.

*Dependability* was assured by the audio-recorded data from the FGDs being transcribed verbatim. Transcripts were reviewed for errors by the co-authors. Debriefing sessions amongst the authors ensured that the process of data collection and data analysis and the results of our study were accurate and that the findings of the FGDs were supported by the data collected.

*Confirmability* was achieved by an audit trail kept for the data collection, data analysis and interpretation of the data. Time was spent with the participants to understand them by checking and rechecking the information that they provided. They were encouraged to speak freely without judgement and were given the opportunity to refuse to participate in our study. To ensure integrity in reporting, there was no data manipulation in interpreting the data in the way the authors desired; the data presentations were the true views of the CHWs.

*Transferability* was achieved by documenting and providing sufficient detail of all the procedures used for covering the recruitment of participants, how the FGDs were conducted and the decisions and conclusions that were made during data analysis, so that other researchers may be able to transfer the results to other contexts or settings.

*Reflexivity*: Although the first author is a physiotherapist, which influenced her to undertake our study, she had no affiliation to the study population and the study population was not aware of her profession. A reflexive journal was kept recording our study process.

### Ethical considerations

Institutional ethical clearance was obtained from the Sefako Makgatho Health Sciences University Research and Ethical Committee (reference number: SMUREC/H/49/2019: PG) and the Tshwane Research Committee (reference number: GP 201907_023). Permission to conduct our study was obtained from the PHC Manager for Tshwane Provincial Clinics.

Participation was voluntary, and potential participants signed informed consent. The FGDs made use of fictitious names to link the demographic data with the data obtained from the participants.

## Findings

### Demographic information of participants

A total of 58 participants took part in the six FGDs. As indicated in Table 1, the teams consisted mainly of female participants (90%), the majority (62%) being younger than 40 years of age, and nearly half (46%) had more than 6 years of working experience as members of WBOTs. The team members were CHWs only, with 3–10 years of working experience in the WBOTs.

**TABLE 1:** Sociodemographic characteristics of participants (*n* = 58).

Characteristics	Frequency	%	Range	Median	SD
**Gender**					
Male	6	10	-	-	-
Female	52	90	-	-	-
**Age in years**			23–60	36	8.8901
23–29	8	14	-	-	-
30–34	16	27	-	-	-
35–39	12	21	-	-	-
40–45	10	17	-	-	-
46–52	9	15	-	-	-
52–60	3	6	-	-	-
**Number of years working as a member of a WBOT (years)**			3–10	5	1.6087
3	13	23	-	-	-
4	2	3	-	-	-
5	16	28	-	-	-
6	22	38	-	-	-
7	2	3	-	-	-
9	1	2	-	-	-
10	2	3	-	-	-

WBOT, Ward-based outreach team.

### Themes and subthemes identified

From the FGD, three themes were identified related to the views of CHWs on the integration of a physiotherapist as a member of a WBOT. The three themes were given as follows:

1. Caring for patients with chronic conditions and disabilities is challenging for WBOTs.
2. People in the community experience poor access to physiotherapy services.
3. The WBOTs can benefit from integrating a physiotherapist.

The first two themes describe the challenges of the WBOTs and the challenges of the community related to physiotherapeutic services, whilst the third theme covers the views of the CHWs on the integration of a physiotherapist into the WBOTs. A summary of the three main themes and eight subthemes that emerged from the data is presented in Table 2.

**TABLE 2:** Themes and subthemes.

Theme		Subtheme
1.	Caring for patients with chronic conditions and disabilities is challenging for WBOTs	1.1 WBOTs lack the rehabilitation knowledge and skills to attend to community needs 1.2 WBOTs lack resources to perform home-based care 1.3 WBOTs are not taken seriously by the community
2.	The community experiences poor access to physiotherapy services	
3.	The WBOT can benefit from integrating a physiotherapist	3.1 Capacity building 3.2 Improved access to rehabilitation services 3.3 Improved follow-up 3.4 Enhanced credibility for WBOTs 3.5 Enhanced WBOT performance

WBOT, Ward-based outreach team.

## Caring for patients with chronic conditions and disabilities is challenging for ward-based outreach teams

Data show that WBOTs experience challenges when managing patients with limited function who need physiotherapeutic services. The WBOTs said that they felt incompetent regarding the quality of care they offered to the community because of their limited rehabilitation knowledge and skills, and they lacked the resources to perform home-based duties as expected. The CHWs felt disrespected in the community.

### Ward-based outreach team lack rehabilitation knowledge and skills to attend to community needs

The WBOTs knew that they were not able to help the community when they needed physiotherapy services as they were not trained, and they lacked the skills of a physiotherapist, as expressed:

‘We don’t know what to do most of the times [*when having to treat a patient who needs physiotherapy services*].’ (Female D, CHW)

‘Lack of knowledge – I think to a certain degree people look at the skills that CHW possesses. They’ll come to a household and say, “this one is not able to assist.” Because they are aware of the limitations of the CHW.’ (Male T, CHW)

‘Most of us are not trained to do what the physio does.’ (Male N, Outreach Team Leader [OTL])

‘With my little knowledge, I can do the exercise, but we need more.’ (Female A, CHW)

### Ward-based outreach teams lack resources to perform home-based care

The participants reported that the WBOTs were not able to meet all the service needs of the community because of a lack of transport, assistive devices, gloves and surgical masks that were needed to assist patients and to perform their tasks:

‘The wheelchair, they are not many – maybe we have got five clients, but only three wheelchairs; we must borrow the wheelchair from the other one for that person to go to the physio.’ (Female K, CHW)

‘So when we walk in, we found that it’s a TB patient lying on bed; we don’t have masks, we don’t have gloves and we just walk to see the patient.’ (Female F, CHW)

### Ward-based outreach teams are not taken seriously by the community

The participants also stated that the WBOTs were not taken seriously by the community:

‘Ahh, they [*community*] just think we are just walking around; we are doing nothing.’ (Female L, CHW)

‘But it differs from person to person; some are willing to go through the process with you. So these are some of the challenges. Being undermined.’ (Male T, CHW)

### Community members experience poor access to physiotherapy services

Access to physiotherapy services was a challenge for community members because of the lack of assisted devices (wheelchairs), inaccessible transport, financial constraints and shortage of physiotherapists in the community:

‘Sometimes we have to take them with their wheelchairs; it is very stressful to see a CHW on the road, main road, maybe during windy days or rainy days walking and taking the client to clinic X or to the hospital for the physiotherapy.’ (Female L, CHW)

‘Like in my area, there is a child [*who needs physiotherapy services*] and they are struggling to go to the clinic. Sometimes the mother doesn’t have the money. The mother is also disabled.’ (Female T, CHW)

‘There is another household in my area [*in*] which granny is using a walking frame. Ahh, she is the breadwinner because of the social grants she receives, so already it doesn’t cover the whole household. Sometimes they sleep hungry. So we cannot force the person to take last money to go to hospital. Although you want that person to be fine again, and you don’t know what caused the problem, and I don’t know what to do.’ (Female L, CHW)

‘The wheelchair, they are not many – maybe we have got five clients, but only three wheelchairs; we must borrow the wheelchair from the other one for that person to go to the physio.’ (Female K, CHW)

‘The physiotherapy appointment queue is long; he has to wait for months to get physiotherapy assistance, and he has to hire a transport to take to that facility as well.’ (Female L, CHW)

‘We are having one physiotherapist who is servicing the whole region that we are dealing with.’ (Male T, CHW)

### The ward-based outreach team can benefit from integrating a physiotherapist

The CHWs believed the inclusion of a physiotherapist as part of the team would be beneficial for them for various reasons, as explained below.

#### Capacity building

A physiotherapist as a member of the WBOTs can provide education and training to other WBOT members:

‘I think the physio will even educate us as community health workers on how to deal with cases that we don’t know how to deal with, that we are not equipped right now to deal with.’ (Male N, OTL)

‘I think they can give us training. I think maybe if they can just give us training so that we can do it at homes.’ (Female G, CHW)

‘Maybe some other times when we are going around with the physiotherapist, he will teach us how to handle the patient with this kind of situation. Even us, we are going to get knowledge from that physio.’ (Female L, CHW)

‘The physiotherapist can also teach us what we can do to our community members. If we come across those challenges, we will also benefit. I think it will be good for all of us.’ (Female O, CHW)

#### Improved access to rehabilitation services

Integrating physiotherapy services in WBOTs will improve access to rehabilitation care:

‘If a physiotherapist is part of this team, people will be able to get help faster than now, quicker.’ (Male N, OTL)

‘Yeah, I think we would like to have the physiotherapy in the WBOT team to go with us to the household to see patients who are vulnerable and can’t walk, those who have challenges.’ (Female F, CHW)

‘The need that we have here at clinic X is that we find older people having a stroke. So they struggle to walk. So I think a physiotherapist must be there to help the older people.’ (Female L, CHW)

‘I think that will reduce queues from the hospitals and also at the clinic; we don’t know how many people are working there. Maybe it’s one seeing the whole of Hammanskraal. So it will be beneficial for them.’ (Female R, CHW)

#### Improve follow-up

Integrating physiotherapists in the WBOTs can improve patient follow-up care:

‘If we do have a therapist in our area, we won’t have people who are lost to follows [*lost to follow-up*], people who are not honouring their appointments.’ (Female L, CHW)

#### Enhance credibility of ward-based outreach teams

The presence of a physiotherapist as part of the WBOT will benefit the community and be of help to the WBOTs to perform their duties:

‘If we can have a physiotherapist based in our clinic, it can be an advantage for us, because we have many clients that need the physiotherapy at this present moment.’ (Female L, OTL)

‘Maybe some other times when we are going around with the physiotherapist, he will teach us how to handle the patient with this kind of situation.’ (Female L, CHW)

‘I think it will be great for us as community health workers to have the physiotherapy. Even if we go to the household, I think will help us to know to gain experience for us to able to help our client.’ (Female T, CHW)

#### Enhance ward-based outreach team performance

The WBOTs were of the view that the integration of a physiotherapist in the team will be able to improve the lives of the people by providing much-needed physiotherapy services:

‘It will reduce the number of those people who are bedridden who are at home.’ (Male C, CHW)

‘The need that we have here at this clinic is that we find older people having a stroke. So they struggle to walk. So I think a physiotherapist must be there to help the older people.’ (Female L, CHW)

## Discussion

Our study explored the views of CHWs on the integration of physiotherapists into WBOTs and found that caring for patients with chronic conditions and disabilities is challenging for the WBOTs. The WBOTs felt insecure and helpless when dealing with patients in the community. They expressed limited health knowledge and limited rehabilitation skills. The limited rehabilitation skills amongst the WBOTs was also noted by Khuzwayo and Moshabela (2017) and Tsolekile et al. (2014). Ward-based outreach teams were of the opinion that their interventions were limited and not always beneficial to those who needed them. WBOTs complained of walking long distances daily, which made them tired by the time they arrived at patients’ homes. These findings are similar to Austin-Evelyn et al. (2017), who found that there was a lack of transport, equipment and limited medical supplies amongst WBOTs. The CHWs who are part of the WBOTs faced negative perceptions in the community, and this was also noted by White, Govender and Lister (2017).

There is poor access to physiotherapy services. Patients need physiotherapy intervention in their homes or at their local clinics, as they could not afford the costs associated with travelling to clinics or hospitals further away. They were referred to the only clinic, clinic X, which had a limited number of physiotherapists. Patients were often given another appointment when they went to clinic X for the first time, as it was not their appointment date. This often delayed their treatment sessions or made patients miss their treatment, as they did not have the money for transport for the next appointment date. This is similar to Vergunst et al. (2015) stating that people with disabilities have limited access to health care because of financial issues and transportation difficulties in reaching health care facilities. Eide et al. (2015) in a study in four African countries confirmed that patients with disabilities face more barriers in accessing health care services and that the socio-economic status of the individuals was the strongest predictor of accessibility to such facilities. The CHWs felt that integrating a physiotherapist as a member of the WBOTs could benefit the health of patients because of the limitations they currently experienced in accessing physiotherapy services on time, that is, lack of money and transport, the poor referral system and the limited number of physiotherapists at clinic X.

The participants reported that the integration of a physiotherapist in WBOTs would improve their confidence, as they would be educated by the physiotherapist on how to handle patients and continue to practise the skills learned. Visagie and Swartz (2016) were of the opinion that one-on-one therapy is not the solution to the rehabilitation needs of persons with disabilities in remote, rural settings, recommending that the rehabilitation in a rural setting should be supported with a transdisciplinary team, family members, CHWs and peer mentors. The benefits of this community-based rehabilitation approach would include cost-effectiveness, less intrusion in family units, holistic service delivery and greater opportunities for service deliverers, such as CHWs on the WBOTs being able to enhance their knowledge and skills (Visagie & Swartz 2016), where skills would be shared and interventions provided across professional boundaries (King et al. 2009; Sims, Hewitt & Harris 2015).

The authors agree with the CHWs on the WBOTs who also indicated that patients would then receive regular services, which would improve their physical outcomes because of more focused interventions provided by the transdisciplinary team. They would be able to get physiotherapy-related services at home and thereby decrease the waiting periods at clinic X, leading to a quicker provision of health services (Hanass-Hancock et al. 2015). The inclusion of a physiotherapist as a member of the WBOTs would play an important role in ensuring universal health coverage for the population of South Africa (Tiwarii, Nedii & Chikte 2020), as per adoption of the NHI plan (South African Government 2022).

## Recommendations

Our study should be repeated in other health regions, which could have wider recommendations if the same findings are repeated. Our study may influence the training of the WBOTs to include basic physiotherapy techniques in their study curriculum so that they have basic rehabilitation skills to be able to manage patients with disabilities. If broadened to include other study sites, our results may influence health policy to include rehabilitation into primary health care.

## Limitations

The main disadvantage of the qualitative approach of our study is that the findings cannot be extended to wider populations with the same degree of certainty that quantitative analyses can.

## Conclusion

Although WBOTs are doing beneficial work in their communities by providing a range of services, they lack physiotherapy services, which disadvantage the community and people with disabilities specifically. In South Africa, the South African Constitution and the ratification of the United Nations Convention on the Rights of Persons with Disabilities (Sherry 2015) support the right to health for people with disabilities. It is thus concluded that policymakers consider the inclusion of a physiotherapist in the WBOTs in the National Department of Health.
